# Food Protein-Induced Enterocolitis Syndrome Causing Hypovolemic Shock in Infants With Down Syndrome

**DOI:** 10.7759/cureus.18366

**Published:** 2021-09-28

**Authors:** Akihiro Iguchi, Yoshihiro Aoki, Katsuhiko Kitazawa

**Affiliations:** 1 Department of Pediatrics, Asahi General Hospital, Chiba, JPN; 2 Department of Emergency and Critical Care Medicine, Aizawa Hospital, Nagano, JPN

**Keywords:** resuscitation, shock, metabolic acidosis, acute renal failure, down syndrome

## Abstract

This study aimed to investigate the clinical characteristics of severe food protein-induced enterocolitis syndrome (FPIES) in patients with Down syndrome. We report the cases of three infants with Down syndrome who were diagnosed with FPIES. All patients presented with hypovolemic shock, metabolic acidosis, and acute kidney injury after introducing a milk-based formula. They required aggressive fluid resuscitation and alternative nutrition. All three patients survived without any complications after the treatment. FPIES may cause hypovolemic shock in infants with Down syndrome and these patients need prompt fluid resuscitation.

## Introduction

Food protein-induced enterocolitis syndrome (FPIES) is a non-immunoglobulin E (IgE) mediated food allergy that develops during infancy [1]. According to the previous international consensus guidelines, while digestive symptoms such as vomiting, diarrhea, and bloody stools are the main symptoms of FPIES, approximately 15% of the FPIES patients also develop hypovolemic shock [2]. Therefore, it is essential for general pediatricians to understand the management of patients with FPIES. The number of reports that discuss severe FPIES in Down syndrome patients is limited, and actual patient management in the acute phase, including fluid resuscitation and nutrition, is not well established. To illustrate the difficulty in the management of severe FPIES in Down syndrome, we report the cases of three infants with Down syndrome who presented with severe FPIES complicated with a hypovolemic shock that required aggressive fluid resuscitation and alternative nutrition during hospitalization.

## Case presentation

Case one

An 18-day-old boy with Down syndrome and no known cardiac anomalies was admitted to our pediatric department owing to poor weight gain. His original nutritional intake exclusively consisted of milk-based formula. After he was hospitalized, several instances of steatorrhea were noticed. His nutrition was changed to an amino acid-based formula on day 34 of hospitalization, and his body weight gradually increased thereafter. On day 85, a partially hydrolyzed formula was administered to improve the taste. However, the patient developed severe diarrhea on the same day, which persisted after the hydrolyzed formula was discontinued. By day 88, he had developed poor sucking, lethargy, and peripheral coldness, and had lost 18% of his body weight within three days. His body temperature, heart rate, systolic blood pressure, and respiratory rate were 37.9°C, 186/min, 118 mmHg, and 50/min, respectively. He developed compensated hypovolemic shock, which required intensive intravenous fluid resuscitation (100 mL/kg) for stabilization. He also developed severe metabolic acidosis and acute renal failure (Table 1). Milk was eliminated from his diet, and his digestive symptoms resolved completely within two days. Despite the absence of vomiting in his entire course, he was provisionally diagnosed with FPIES considering the results of the elimination test, oral food challenge, and allergen-specific lymphocyte stimulation test (ALST) (Table 1). After three days of fasting, we reintroduced the amino acid-based formula on day 92. As his body weight steadily increased, he was discharged on day 117.

**Table 1:** Characteristics of three infants with Down syndrome complicated by food protein-induced enterocolitis syndrome TAM: transient abnormal myelopoiesis, PFO: patent foramen ovale, SI: stimulation index, ALST: allergen-specific lymphocyte stimulation test, NA: not applicable

	Reference Range	Case 1	Case 2	Case 3
Age (months)		2	1	5
Sex		boy	girl	girl
Complications of Down syndrome		TAM, PFO	none	Hirschsprung disease
Rotavirus vaccine		none	none	none
Digestive symptoms		watery diarrhea	vomiting, watery diarrhea	vomiting, watery diarrhea
Loss of weight (%)		-18	-21	-7.0
Identification of shock by severity		compensatory	hypotensive	hypotensive
Blood test results				
pH	7.35–7.45	7.02	6.89	7.12
bicarbonate (mmol/L)	20.0–26.0	7.1	5.4	8.5
base excess (mmol/L)	-3.0–3.0	-22.2	-27.0	-19.4
lactate (mg/dL)	4.5–18.0	28.1	13.6	37.9
blood urea nitrogen (mg/dL)	6.0–20.0	92.0	21.0	25.0
serum creatinine (mg/dL)	0.14–0.26	2.54	0.94	1.20
serum sodium (mEq/L)	135–145	170	150	126
serum pottasium (mEq/L)	4.0–5.5	4.9	3.6	5.1
serum chloride (mEq/L)	98–108	144	139	105
C-reactive protein (mg/dL)	< 0.15	0.10	3.6	19.5
white blood cell (/μL)	6.0–17.5 × 10^3^	18.0 × 10^3^	32.6 × 10^3^	22.2 × 10^3^
eosinophil (%)	0.2-8.4	1.0	2.0	0.2
haemoglobin (g/dL)	9.5–13.7	9.5	11.9	13.0
platelet (/μL)	15.0–40.0×10^4^	47.1×10^4^	34.4×10^4^	26.4×10^4^
Blood culture	negative	negative	negative	negative
Fecal test				
occult blood	negative	negative	negative	negative
eosinophils	negative	negative	negative	negative
rotavirus antigen	negative	NA	negative	negative
adenovirus antigen	negative	NA	negative	negative
Fecal culture	negative	negative	negative	negative
SI in the patient's ALST (%)	< 180	1688	346	NA
Elimination test	negative	positive	positive	positive
Total amount of fluid used for resuscitation		100 mL/kg	120 mL/kg	60 mL/kg
Antibiotic use		none	cefotaxime	cefotaxime
Hospitalization (days)		117	16	15
Outcome		Survived	Survived	Survived

Case two

A one-month-old girl with Down syndrome and no known cardiac anomalies, whose diet comprised milk-based formula, visited our outpatient clinic with vomiting and chronic diarrhea. Three days before admission, she showed poor sucking. On admission, she presented with 21% weight loss, an impalpable radial pulse, systemic cyanosis, and poor skin turgor. Her body temperature, heart rate, systolic blood pressure, and respiratory rate were 36.5°C, 120/min, 58 mmHg, and 40/min, respectively. She developed hypotensive hypovolemic shock and required intravenous fluid resuscitation at 120 mL/kg. Blood tests on admission revealed a high white blood cell count, which led us to consider septic shock as a differential diagnosis; thus, cefotaxime (CTX) was initiated (Table 1). She also developed severe metabolic acidosis and acute renal failure. Blood, urine, and stool cultures were negative; therefore, CTX was discontinued on hospitalization day six. Elimination of milk from her diet effectively improved her symptoms. Thus, based on her clinical course, she was diagnosed with FPIES. On day five of hospitalization, we initiated an amino acid-based formula after four days of fasting. Subsequently, a partially hydrolyzed formula was introduced on day 12. She was discharged on day 16 without any digestive symptoms. 

Case three

A five-month-old girl with Down syndrome and no known cardiac anomalies on a diet of breast milk presented to our outpatient clinic with vomiting, diarrhea, and lethargy. The day before admission, she was fed infant formula milk for the first time. One hour later, she vomited and passed a large amount of watery diarrhea twice. On admission, she presented with 7% weight loss, dry oral mucosa, peripheral cyanosis, and a prolonged capillary refill time (over 5 s). Her body temperature, heart rate, systolic blood pressure, and respiratory rate were 36.9°C, 200/min, 60 mmHg, and 42/min, respectively. She developed hypotensive hypovolemic shock due to severe dehydration and required intravenous fluid resuscitation at 60 mL/kg. Based on her vital signs and high white blood cell count, we suspected septic shock, and CTX was administered (Table 1). Furthermore, she experienced severe metabolic acidosis and acute renal failure. Blood, urine, and stool cultures were negative. Therefore, CTX was discontinued after six days. She was diagnosed with FPIES based on her clinical course and improvement in digestive symptoms after milk was eliminated from her diet. On hospitalization day two, breastfeeding was resumed, and her diarrhea later improved. We also introduced an amino acid-based formula, in case there was a shortage of breast milk at home, and confirmed that no digestive symptoms occurred secondary to formula administration. She was discharged on day 15.

## Discussion

We described the cases of three infants with Down syndrome who developed severe FPIES owing to milk-based formula. In children with Down syndrome, interleukin-10 levels are lower and tumor necrosis factor-α levels are higher than in those without it [3]. Wakiguchi et al. [4] reported that FPIES in Down syndrome patients was worse due to immunological impairment, such as lower serum interleukin-10 levels. They also reported a case of severe FPIES in a Down syndrome patient who developed severe diarrhea, acute kidney injury, and metabolic acidosis, as in our cases. As FPIES with diarrhea worsens progressively [5], when infants with Down syndrome develop digestive symptoms after consuming a milk-based formula, FPIES should be considered as a possible diagnosis.

It is common for delayed diagnosis to occur with FPIES [6]. In case one, although FPIES was suspected during hospitalization, diarrhea secondary to moderately hydrolyzed formula administration persisted for two days, despite immediate formula discontinuation. Furthermore, in case three, the patient also presented with severe hypotensive shock despite arriving at the hospital one day after diarrhea onset. Therefore, clinicians should know that FPIES during infancy can progress rapidly. Acute gastroenteritis, sepsis, metabolic disorders, and intussusception are differential diagnoses for FPIES [7]. Differentiating FPIES from acute gastroenteritis is particularly important (as acute gastroenteritis is common in general pediatrics) because the symptoms may worsen if the original milk-based formula is administered continuously. 

Down syndrome is difficult to manage in the emergency department due to various associated complications. Regarding the shock status, the correct type of shock must be identified by reviewing the past medical history, presenting illness, and clinical findings. As the congenital heart disease prevalence among Down syndrome patients is 50-60% [8], cardiogenic shock is a differential diagnosis, although it is rarely encountered in general pediatric practice. None of our cases had congenital heart disease and all had a medical history of frequent diarrhea, concerning weight loss, and obvious physical signs of dehydration that led to a hypovolemic shock diagnosis. While most patients with FPIES are afebrile, some can develop fever or present with a high white blood cell count, mimicking sepsis [1, 2]. In cases two and three, we initiated CTX because we could not rule out the possibility of septic shock on admission. In the relevant situations, prompt fluid resuscitation must be prioritized before determining the cause of hypovolemia.

Limitations

This retrospective review included only three cases with Down syndrome complicated by severe FPIES. Furthermore, according to the international consensus guidelines [2], the diagnosis of case one could not be confirmed as FPIES owing to the absence of vomiting. However, based on elimination of other differential diagnoses [2], the occurrence of severe diarrhea after administering a partially hydrolyzed formula, and the positive result of ALST [9], FPIES was strongly suspected.

## Conclusions

FPIES should be suspected in Down syndrome infants with digestive symptoms following milk-based formula ingestion. Down syndrome patients with FPIES may deteriorate rapidly. In Down syndrome, severe FPIES is a possible cause of hypovolemic shock which requires prompt fluid resuscitation.
